# Effects of acetaminophen on mitochondrial complex I activity in the rat liver and kidney: a PET study with ^18^F-BCPP-BF

**DOI:** 10.1186/s13550-016-0241-4

**Published:** 2016-11-21

**Authors:** Hiroyuki Ohba, Masakatsu Kanazawa, Takeharu Kakiuchi, Hideo Tsukada

**Affiliations:** Central Research Laboratory, Hamamatsu Photonics K.K., 5000 Hirakuchi, Hamamatsu, Shizuoka 434-8601 Japan

**Keywords:** Hepatitis, Nephritis, Acetaminophen, Mitochondria complex I, PET

## Abstract

**Background:**

In the present study, 2-tert-butyl-4-chloro-5-[6-(4-^18^F-fluorobutoxy)-pyridin-3-ylmethoxy]-2H-pyridazin-3-one (^18^F-BCPP-BF), a PET probe for mitochondrial complex I (MC-I), was used to validate whether MC-I is a useful biomarker for detecting acetaminophen-induced dysfunctions in the liver and kidney.

The kinetic and distribution of ^18^F-BCPP-BF were assessed in rats using high-resolution animal PET in vivo. The binding specificity of ^18^F-BCPP-BF to MC-I in the liver and kidney was confirmed by the pre-administration of rotenone, a specific MC-I inhibitor. The effects of acetaminophen on MC-I activity were assessed 2 and 24 h after the administration of vehicle or acetaminophen at a dose of 100 or 300 mg/kg. Biochemical parameters in plasma and urine were assessed 2, 6, and 24 h after the administration of vehicle or acetaminophen.

**Results:**

The uptake of ^18^F-BCPP-BF by the liver and kidney was significantly inhibited by the pre-administration of rotenone. Two and more hours after the administration of acetaminophen, the uptake of ^18^F-BCPP-BF was dose-dependently reduced in the liver, even at 100 mg/kg, and in the kidney at 300 mg/kg, whereas biological parameters started to be affected 6 h or later at doses of 300 mg/kg.

**Conclusions:**

The present study demonstrated that ^18^F-BCPP-BF has potential as a PET probe for the quantitative imaging of hepatic and renal dysfunction as impaired MC-I activity in the early phase of the treatment for an overdose of acetaminophen in the living body with PET.

## Background

Mitochondria are present in every mammalian cell except red blood cells and provide the majority of energy in the form of adenosine triphosphate (ATP). Tissues with high energy demands, such as the brain, heart, muscle, liver, and kidney, contain the greatest number of mitochondria. The electron transport chain in mitochondria, which consists of five complexes from I to V, is the major site of ATP production. Among these complexes, the complex I (MC-I) is the first and rate-limiting step of the overall respiratory activity and oxidative phosphorylation under physiological conditions. Mitochondrial dysfunctions contribute to the pathophysiologies of many acute and chronic diseases. Mitochondria are also regarded as the main intracellular source of reactive oxygen species (ROS) in cells as well as a main target of ROS-mediated damage.

Acetaminophen is a widely used antipyretic and analgesic that is safe and effective when used at therapeutic dose ranges; however, an overdose of acetaminophen induces the etiology of acute hepatotoxicity [1, 2] and nephrotoxicity [3]. The first reports of acetaminophen hepatotoxicity in humans published in the 1960s [4] and acetaminophen overdoses have since been identified as the most common cause of acute liver failure in Western countries. In the USA, an overdose of acetaminophen is responsible for approximately 56,000 emergency room visits, 26,000 hospitalizations, and nearly 500 deaths each year [5].

The therapeutic dose range of acetaminophen is glucuronidated or sulfated for excretion, and its small amount is metabolized to *N*-acetyl-p-benzoquinone imine (NAPQI), a chemically reactive intermediate, by oxidation via cytochrome P450 2E1 (CYP2E1) [6]. At therapeutic doses, NAPQI is readily detoxified by conjugation with glutathione, whereas an overdose of acetaminophen saturates the glucuronidation pathway. The resulting excess amount of NAPQI was previously assumed to react covalently with the sulfhydryl groups of cysteine in glutathione and several proteins and decreases glutathione levels, thereby contributing to increased oxidative/nitrative stress [7].

However, recent studies revealed that the oxidative modification of thiol molecules by NAPQI was minimal in response to acetaminophen [8–11]. The immunoprecipitation of mitochondrial superoxide dismutase (MnSOD), a critical enzyme in the detoxification of ROS, from the livers of acetaminophen-treated mice did not detect acetaminophen-MnSOD adducts [9]. These findings suggest that the production of NAPQI is not the only reason for acetaminophen-induced liver necrosis and also that CYP2E1 may have a limited role in the formation of NAPQI at higher doses of acetaminophen [10].

Alternatively, acetaminophen has been reported to promote liver and kidney injury through enhanced protein nitration due to increased levels of the reactive nitrogen species, nitric oxide and peroxynitrite, which are formed through the rapid reaction of superoxide with nitric oxide [9, 11, 12]. Peroxynitrite is a nitrating and oxidizing agent and is detoxified by glutathione [13]. Nitrated proteins in mitochondria, such as aldehyde dehydrogenase, ATP synthase, 3-ketoacyl-CoA thiolase, MnSOD, and glutathione peroxidase, play a role in promoting acetaminophen-induced mitochondrial dysfunction in the liver [12]. Of these proteins, MnSOD in the mitochondrial matrix detoxifies the superoxide radical, and this is followed by the formation of hydrogen peroxide and oxygen. Therefore, MnSOD limits the reaction of superoxide with nitric oxide to form the reactive nitrogen species peroxynitrite. Although the effects of the nitration of proteins are poorly understood at the molecular level, acetaminophen may lead to a decrease in ATP synthase [10] and MnSOD [9–11] activities through the nitration of Tyr residues on their active sites [14]. The nitration of critical Tyr residues in these enzymes results in their damage as well as mitochondrial dysfunctions through the inactivation of these activities by conformational changes [15]. In addition, hepatotoxicity induced by peroxynitrite was found to be markedly attenuated by the anti-oxidant glutathione [16] and *N*-acetylcysteine (NAC), which functions by increasing glutathione levels and binding with the reactive metabolite of acetaminophen [16, 17]. Nitrative modifications to critical enzymes 2 h after the administration of acetaminophen appear to contribute to mitochondrial dysfunctions, resulting in severe necrosis 24 h after its administration [10]. Most organs may be affected in mitochondrial cytopathies that primarily affect oxidative phosphorylation. From the renal point of view, since podocytes with abundant mitochondria have limited potency for regeneration, mitochondrial cytopathies induce irreversible podocyte damage and microvascular lesions, which comprise a fatal strike to kidney function. These findings suggest that the non-invasive assessment of mitochondrial activity has potential as a useful pathophysiological index for the prediction and diagnosis of hepatitis and nephritis.

We recently developed novel PET probes with different lipophilicity and affinity to MC-I, 2-tert-butyl-4-chloro-5-{6-[2-(2-^18^F-fluoroethoxy)-ethoxy]-pyridin-3-ylmethoxy}-2H-pyridazin-3-one (^18^F-BCPP-EF; logD_7.4_ = 3.03; Ki = 2.31 nM), 2-tert-butyl-4-chloro-5-[6-(4-^18^F-fluorobutoxy)-pyridin-3- ylmethoxy]-2H-pyridazin-3-one (^18^F-BCPP-BF; logD_7.4_ = 4.27; Ki = 0.70 nM), and 2-tert-butyl-4-chloro- 5-{6-[2-(2-^11^C-methoxy-ethoxy)-ethoxy]-pyridin-3-ylmethoxy}-2H-pyridazin-3-one (^11^C-BCPP-EM; logD_7.4_ = 2.87; Ki = 4.70 nM) for the quantitative imaging of MC-I in vivo [18, 19]. The capability of ^18^F-BCPP-EF to detect neuronal damage as impaired MC-I activity has already been evaluated in the brains of ischemic stroke [19, 20] and Parkinsonism [21] animal models, as well as in the brains of aged monkeys [22, 23]. Besides the brain and heart, kinetic analyses using the tissue dissection method have also indicated the strong uptake of these PET probes in the liver and kidney of the rat [19].

In the present study, the capability of PET imaging using ^18^F-BCPP-BF to detect acetaminophen-induced changes in hepatitis and nephritis in the rat was evaluated. The kinetic and distribution of ^18^F-BCPP-BF were assessed using high-resolution animal PET in vivo. The binding specificity of ^18^F-BCPP-BF to MC-I in the liver and kidney was confirmed by the pre-administration of rotenone, a specific MC-I inhibitor. The effects of acetaminophen on MC-I activity were assessed 2 and 24 h after the administration of vehicle or acetaminophen at a dose of 100 or 300 mg/kg. These PET imaging data in the liver and kidney were further compared with the conventional urinal and serum biochemical parameters of the liver and kidney of rats treated with acetaminophen.

## Methods

### Animals and chemicals

The following experiments were approved by the Ethical Committee of the Central Research Laboratory, Hamamatsu Photonics. Control male Sprague-Dawley rats (8 weeks of age; 260–280-g body weight) were purchased from Japan SLC, Inc. (Hamamatsu, Japan). Acetaminophen was from Wako Pure Chemical Industry (Osaka, Japan). Isoflurane and rotenone were purchased from Dainippon Pharmaceutical (Osaka, Japan) and MP Biochemicals LLC (Illkirch, France), respectively. The precursor of ^18^F-BCPP-BF and its standard compound were obtained from the NARD Institute (Amagasaki, Japan).

### PET ligand synthesis

Positron-emitting fluorine-18 (^18^F) was produced by the ^18^O(p, n)^18^F nuclear reaction using the cyclotron (HM-18, Sumitomo Heavy Industry, Ltd., Tokyo, Japan) at Hamamatsu Photonics PET center. Labeled compounds were synthesized using a modified CUPID system (Sumitomo Heavy Industry, Ltd., Tokyo, Japan). HPLC analyses of the labeled compounds were performed on a GL-7400 low-pressure gradient HPLC system (GL Sciences, Inc., Tokyo, Japan) with a radioactivity detector (RLC-700, Hitachi Aloka Medical, Inc., Tokyo, Japan).

^18^F-BCPP-BF was radiolabeled by nucleophilic ^18^F-fluorination of the corresponding precursor as reported previously [18–23]. The radiochemical purity and specific radioactivity of ^18^F-BCPP-BF was more than 99% and 64.7 ± 25.3 GBq/μmol, respectively.

### PET measurements

Four animals per group were used for PET imaging. In order to evaluate the specific binding of ^18^F-BCPP-BF to MC-I in the liver and kidney, vehicle or rotenone, a specific MC-I inhibitor, at a dose of 0.1 mg/kg in 10 mL of vehicle (*N*,*N*-dimethylformamide/polyethylene glycol 400/saline = 1/1/2) was continuously infused into control rats through the tail vein for 1 h, followed by an injection of ^18^F-BCPP-BF into rats through the tail vein.

In order to evaluate the acute effects of acetaminophen, vehicle or acetaminophen at a dose of 100 or 300 mg/kg in 10 mL/kg of vehicle (saline containing 20% Tween-80) was intraperitoneally administered 2 or 24 h before the PET probe injection. Acetaminophen doses were selected based on a previous study [24], which showed that 300 mg/kg was the minimum dose needed to induce toxicity in rats. The time points of PET imaging were selected according to a previous report [10], which revealed that nitrative modifications of mitochondrial enzymes 2 h after the administration of acetaminophen contributed to mitochondrial dysfunctions, resulting in severe necrosis 24 h after its administration.

Rats anesthetized by 1.5–2.0% isoflurane in O_2_ were positioned prone on a fixation plate and placed in the gantry hole of the PET scanner (SHR-7700, Hamamatsu Photonics, Hamamatsu, Japan) [25]. After a transmission scan for 15 min using a ^68^Ge-^68^Ga rotation rod source, ^18^F-BCPP-BF at ca. 15 MBq was intravenously injected into each rat via the tail vein, and this was followed by an emission scan with list mode data acquisition (every 1 ms) for 90 min. The body temperature of each animal was monitored to record throughout the study using a Thermo-Hygro Recorder (TR-72Ui, T&D Corporation, Matsumoto, Japan).

The PET data obtained were reconstructed by Dynamic RAMLA (DRAMA) method with a Gauss filter of 1.0-mm FWHM and attenuation correction using the transmission scan data. Dynamic images every 2 min for the time activity curve (TAC) as well as summation images in the early (from 15 to 30 min after ^18^F-BCPP-BF injection) and late (from 70 to 90 min) phases were reconstructed. Since there were no information on the TACs of ^18^F-BCPP-BF in the liver and kidney, we conducted PET data acquisition up to 90 min after the injection. Then, we assessed which time period was suitable to observe the effects of rotenone, a specific MC-I inhibitor, by using PMOD software (PMOD Technologies Ltd., Zurich, Switzerland). Since the uptake levels of ^18^F-BCPP-BF in these regions were high and relatively stable in early phase (from 15 to 30 min) in control condition, and also the inhibitory effects of rotenone on ^18^F-BCPP-BF uptake showed the comparable results between the early (from 15 to 30 min) and late (from 70 to 90 min) phases, we decided to apply averaged data in early phase (from 15 to 30 min) for the assessments of acetaminophen effects.

Standard uptake value (SUV) images were created as follows:

$$ \mathrm{S}\mathrm{U}\mathrm{V}={C}_{\mathrm{T}}\frac{W_{\mathrm{s}}}{D_{\mathrm{inj}}} $$

where *C*_T_ is the tissue radioactivity (Bq/mL) obtained from PET images, *W*_s_ is the body weight (g) of the rat being examined, and *D*_inj_ is the injected dose (Bq) of ^18^F-BCPP-BF. Each summation SUV image was superimposed on the corresponding X-CT images obtained using ClairvivoCT (Shimadzu Corporation, Kyoto, Japan). Volumes of interest (VOIs) were selected on summation images of the kidney (renal cortex and pelvis) and liver in the early (from 15 to 30 min) and late (from 70 to 90 min) phases and TACs were obtained from dynamic image data in each organ as SUV by setting the early phase VOIs.

### Physiological and biochemical assessments

In a separate study from PET measurements, physiological and biochemical assessments were conducted on four animals for each time and dosing condition. Body weight measurements, urine collection, and blood sampling were conducted 2, 6, and 24 h after the administration of acetaminophen. A rat was placed in a metabolic cage on a 12/12-h light/dark cycle, and urine samples were collected for 24 h and centrifuged to remove suspended material, and the supernatants were analyzed to measure total protein, creatinine, and *N*-acetyl-β-D-glucosaminidase (NAG) using a biochemical automatic analyzer (JCA-BM6010, JEOL Ltd., Tokyo, Japan). Blood sample was collected from the tail vein, and the serum fraction separated from the blood was used in assay of alanine aminotransferase (ALT), aspartate aminotransferase (AST), lactate dehydrogenase (LDH), bilirubin (BIL), and BUN using a biochemical automatic analyzer (JCA-BM6010, JEOL Ltd., Tokyo, Japan).

### Statistical analysis

Results are expressed as means ± SD. The significance of differences was assessed by one-way analysis of variance (ANOVA) followed by Dunnett’s multiple comparison tests. A probability level of less than 5% (*p* < 0.05) was considered to indicate significance.

## Results

**Fig. 1:**
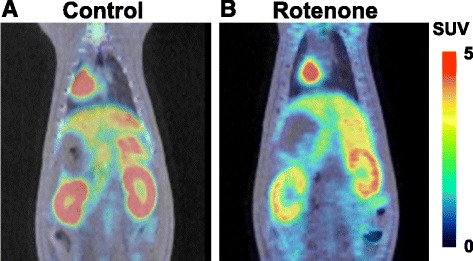
PET-CT images of rat abdominal organs using ^18^F-BCPP-BF. Vehicle or rotenone (0.1 mg/kg) was continuously infused into control rats through the tail vein for 1 h, followed by ^18^F-BCPP-BF injection into rat through the tail vein. PET scanning was conducted for 90 min, summation PET data from 15 to 30 min were reconstructed for SUV images, and PET images were then superimposed on individual X-CT images. **a** Control. **b** Rotenone

**Fig. 2:**
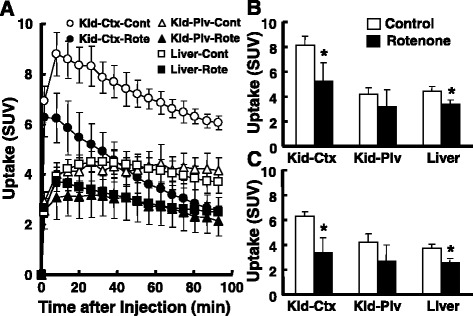
Effects of rotenone on time activity curves (TACs) (**a**) and uptake of ^18^F-BCPP-BF at early (**b**) and late (**c**) phases by the liver and kidney of the control rat. Vehicle or rotenone (0.1 mg/kg) was continuously infused into the control rats through the tail vein for 1 h, followed by the ^18^F-BCPP-BF injection into rats through the tail vein. PET scanning was conducted as described in the legend of Fig. 1. The volumes of interest (VOIs) were set on the kidney (renal cortex and pelvis) and liver, and TACs were obtained in each organ as SUV (**a**). Averaged SUV values from 15 to 30 min (**b**) and from 70 to 90 min (**c**) after the ^18^F-BCPP-BF injection were calculated from the data of time activity curves. *p < 0.05 vs. vehicle

In addition to the liver, PET measurements (Fig. 1a) of ^18^F-BCPP-BF clearly imaged the renal cortex with discrimination from the renal pelvis. The TACs of ^18^F-BCPP-BF demonstrated reversible-type kinetics showing peak uptake in by the renal cortex and liver and constant uptake in the renal pelvis (Fig. 2a), revealing strong uptake by the renal cortex (SUV = 8.12 ± 0.69), moderate by the liver (4.40 ± 0.39) and renal pelvis (4.17 ± 0.51), and weak by the bladder (0.88 ± 0.42, data not shown) as averaged SUV from 15 to 30 min post-injection (Fig. 2b). The uptakes of ^18^F-BCPP-BF calculated as averaged SUV from 70 to 90 min post-injection were 6.29 ± 0.30, 3.75 ± 0.38, and 4.37 ± 0.64 in the renal cortex, liver, and renal pelvis, respectively (Fig. 2c). With the pre-administration of rotenone at a dose of 0.1 mg/kg/h, the uptake level of ^18^F-BCPP-BF calculated as averaged SUV from 15 to 30 min post-injection was markedly reduced in the liver (76.9%/*p* = 0.038 vs. control) and renal cortex (64.2%/*p* = 0.027 vs. control) than in the control (Figs. 1b and 2b). The inhibition degrees calculated as averaged SUV from 70 to 90 min post-injection were 67.9% (*p* = 0.003) and 52.2% (*p* = 0.006) of each control in the liver and renal cortex, respectively (Fig. 2c). Since two kinds of averaged SUV data of ^18^F-BCPP-BF uptake in early (from 15 to 30 min after the injection) and late (from 70 to 90 min) phases showed similar results under control and rotenone conditions, data analyses were conducted using early phase averaged SUV.

**Fig. 3:**
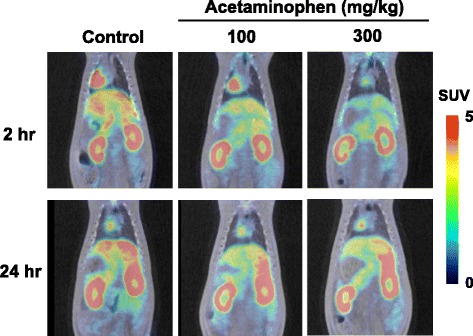
Effects of acetaminophen on the uptake of ^18^F-BCPP-BF in the liver and kidney of the rat. Vehicle or acetaminophen at a dose of 100 or 300 mg/kg was intraperitoneally administered, and PET scanning was conducted using ^18^F-BCPP-BF 2 or 24 h after the administration of acetaminophen. Summation PET data from 15 to 30 min were reconstructed for SUV images, and PET images were then superimposed on individual X-CT images as described in the legend of Fig. 1

**Fig. 4:**
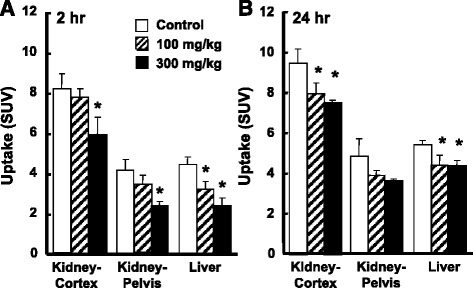
Effects of acetaminophen on the uptake of ^18^F-BCPP-BF in the kidney and liver of rats. Two (**a**) or 24 h (**b**) after the administration of vehicle or acetaminophen at a dose of 100 or 300 mg/kg, PET scans were conducted as described in the legend of Fig. 1. Averaged SUV values from 15 to 30 min after the ^18^F-BCPP-BF injection were calculated as described in the legend of Fig. 2. *p < 0.05 vs. vehicle

As shown in Fig. 3, a dose-dependent reduction in the uptake of ^18^F-BCPP-BF by the liver was induced 2 h after the administration of acetaminophen (72.1%/*p* = 0.017 and 53.9%/*p* = 0.003 vs. control at 100 and 300 mg/kg, respectively) (Fig. 3, upper panel, and Fig. 4a), whereas dose dependency in its uptake was not determined 24 h after the administration of acetaminophen (81.4%/*p* = 0.049 and 80.5%/*p* = 0.002 vs. control at 100 and 300 mg/kg, respectively) (Fig. 3, lower panel, and Fig. 4b). In the renal cortex, the reduced uptake of ^18^F-BCPP-BF was detected 2 h after the administration of acetaminophen at a high dose of 300 mg/kg (72.1%/*p* = 0.025 vs. control) (Fig. 3, upper panel, and Fig. 4a), whereas a significant reduction in its uptake was observed 24 h after the administration of a low dose of 100 mg/kg without dose dependency (83.4%/*p* = 0.038 and 79.5%/*p* = 0.009 vs. control at 100 and 300 mg/kg, respectively) (Fig. 3, lower panel, and Fig. 4b). In the renal pelvis, the reduced uptake of ^18^F-BCPP-BF was detected at 300 mg/kg 2 h after the administration of acetaminophen (56.6%/*p* = 0.027 vs. control) (Fig. 3, upper panel, and Fig. 4a), whereas no significant reduction in its uptake was observed 24 h after (Fig. 3, lower panel, and Fig. 4b).

**Fig. 5:**
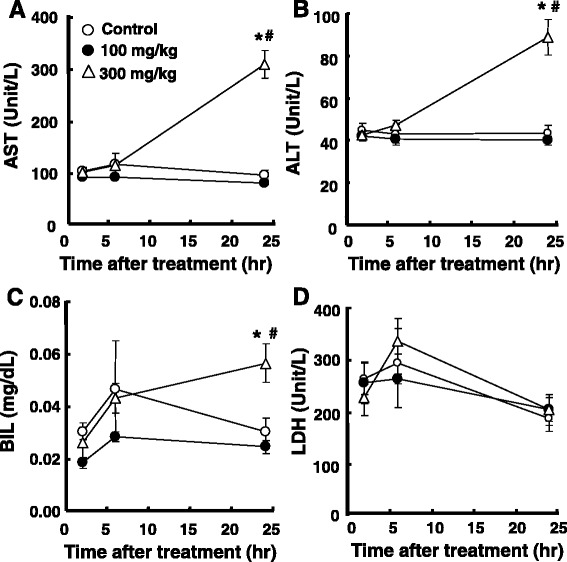
Effects of acetaminophen on biochemical parameters for hepatic function in the rat. Blood samples were collected 2, 6, and 24 h after the administration of acetaminophen, and the serum fraction separated from blood was used for the assay of ALT (**a**), AST (**b**), BIL (**c**), and LDH (**d**). *p < 0.05 vs. vehicle, ^**#**^ *p* < 0.05 vs. 100 mg/kg acetaminophen

Hepatic biochemical parameters indicated that AST, ALT, and BIL significantly increased 24 h after the acetaminophen treatment, but only at a dose of 300 mg/kg (322.3%/*p* < 0.001, 207.4%/*p* = 0.001, and 186.2%/*p* = 0.034 vs. the corresponding control; 383.0%/*p* < 0.001, 224.6.4%/*p* = 0.001, and 233.3%/*p* = 0.034 vs. the corresponding 100 mg/kg), whereas their levels did not show significant changes at a dose of 100 mg/kg (Fig. 5a–c). LDH did not show any significant alterations after the acetaminophen treatment from the control condition (Fig. 5d).

**Fig. 6:**
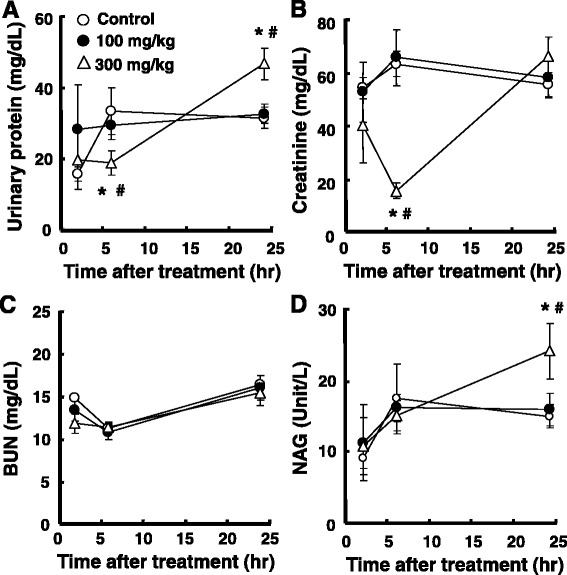
Effects of acetaminophen on biochemical parameters for renal function in the rat. Blood and urine samples were collected 2, 6, and 24 h after the administration of acetaminophen. Urine was centrifuged to remove suspended material and the supernatants analyzed to measure total urinary protein (**a**), creatinine (**b**), and NAG (**d**). Blood samples were collected 2, 6, and 24 h after the administration of acetaminophen, and the serum fraction separated from the blood was used for the assay of BUN (**c**). *p < 0.05 vs. vehicle, ^**#**^ *p* < 0.05 vs. 100 mg/kg acetaminophen

Nephritis biochemical parameters revealed that urinary protein excretion was decreased (56.4%/*p* = 0.028 vs. control; 46.9%/*p* = 0.017 vs. 100 mg/kg) 6 h after and increased (128.9%/*p* = 0.027 vs. control; 143.0%/*p* = 0.027 vs. 100 mg/kg) 24 h after the acetaminophen treatment at a dose of 300 mg/kg (Fig. 6a). Creatinine levels significantly decreased after 6 h at a dose of 300 mg/kg (24.3%/*p* < 0.001 vs. control; 23.4%/*p* = 0.002 vs. 100 mg/kg) and returned to the vehicle level (Fig. 6b). BUN did not show any significant alterations with the acetaminophen treatment at any time (Fig. 6c). NAG showed a significant increase 24 h after the acetaminophen treatment, but only at a dose of 300 mg/kg (161.8%/*p* = 0.011 vs. control; 164.8%/*p* = 0.027 vs. 100 mg/kg), and did not show any significant changes at a dose of 100 mg/kg (Fig. 6d).

## Discussion

The present study demonstrated the capability of PET imaging of MC-I activity using ^18^F-BCPP-BF for the acute phase detection of hepatitis and nephritis induced by an overdose of acetaminophen in rats. The uptake of ^18^F-BCPP-BF by the liver and kidney was significantly reduced as early as 2 h after the administration of acetaminophen, whereas biochemical parameters in plasma and urine started to be affected 6 h or later at the high dose of 300 mg/kg.

The liver and kidney are highly energetic organs and rich in mitochondria, and as expected, the present study demonstrated the uptake of ^18^F-BCPP-BF by these two organs. The strong uptake of ^18^F-BCPP-BF was detected by the kidney, particularly by the renal cortex, while weaker uptake by the renal pelvis was also observed. This stronger renal cortical uptake may be attributed to a disparity in blood flow between the cortex and pelvis in the kidney; the cortical blood flow is fivefold greater than pelvis blood flow [26]. However, this was unlikely because reversible-type kinetics such as ^18^F-BCPP-BF are generally expected to be less sensitive to blood flow changes [27]. We previously reported the low cerebral blood flow dependence of ^18^F-BCPP-EF, an analog of ^18^F-BCPP-BF, with reversible-type kinetics in the living brains of stroke [20] and aged monkeys [22]. In addition, the uptake of ^18^F-BCPP-BF by the kidney as well as the liver was significantly suppressed by the pre-administration of rotenone, a specific MC-I inhibitor, which fully inhibited the binding of MC-I PET probes to MC-I in the heart and brain at a dose of 0.1 mg/kg [19]. We previously reported that dose escalation of more than 0.1 mg/kg was almost impossible because of lethal effects on the cardiac function [19]. Taken together, these results confirmed that the uptake of ^18^F-BCPP-BF reflected MC-I activity, not just blood flow difference, in the liver and kidney.

One of the most interesting findings of the present study was that the acute adverse effects of acetaminophen on the liver and kidney were detectable as the reduced uptake of ^18^F-BCPP-BF as early as 2 h after acetaminophen dosing, whereas the conventional biochemical parameters in plasma and urine commonly used in medical setting started to be affected 6 h or later at the higher dose. These results demonstrated that the non-invasive monitoring of MC-I activity is a useful and valuable index with higher sensitivity for hepatic and renal impairments. ALT and AST have been recognized to reflect liver cell death, while other parameters such as the prothrombin time and bilirubin show temporally losses and the later recovery of liver functions. Proteinuria and serum parameters are still the gold standard in the care of nephritis but are non-specific; they only occur in the later phase of nephritis, and up to 54% of patients with renal allograft rejection do not present these clinical signs [28]. The invasive procedure of biopsy, as the other gold standard, carries the risk of significant organ injury as well as a potential misdiagnosis because of limited sampling sites. Thus, these biomarkers cannot always be used to predict patient outcomes at early time points of disease progression.

Functional imaging of the liver and kidney with PET has been limited. A previous clinical PET study demonstrated that although there was a significant difference in renal blood flow, measured by ^15^O-H_2_O-PET, between healthy subjects and patients with renal dysfunction, renal blood flow was not significantly different in patients with moderate and severe disease [29]. In the molecular imaging of kidney function with PET, the activity of glucose transporters (GLUT1, 3, and 4) has been expected to be assessed using ^18^F-FDG-PET even in the kidney. However, ^18^F-FDG is not appropriate for imaging glucose metabolism in the liver [30] or kidney [31]. The proximal kidney tubules, which compose more than 90% of the renal cortex, rely on fatty acids rather than glucose, which is the substrate of MC-I and MC-III to generate ATP, resulting in the low contribution of glucose utility in the renal cortex (=low ^18^F-FDG uptake). Furthermore, although glucose is completely reabsorbed in the proximal tubules of the kidney, ^18^F-FDG is continuously excreted into the tubular lumen and accumulates along the renal collecting system, inducing strong radioactivity uptake in the kidneys, urinary tract, and bladder. In contrast, its exclusive accumulation in the renal cortex suggests that ^18^F-BCPP-BF in the glomerular filtrate is reabsorbed by active transport in the proximal convoluted tubule. This accounts for the absence of observable urinary tracer activity in the intrarenal collecting system. 2-Deoxy-2-^18^F-fluororibose (^18^F-2-DFR), which accumulates preferentially in the liver through the ribose salvage pathway, has been proposed for the imaging of liver function [30]. Acetaminophen at 300 mg/kg, which is a toxic dose for mice, caused only an approximately 20% reduction in ^18^F-2-DFR uptake by the liver. In contrast, the present study demonstrated that MC-I activity in the liver assessed using ^18^F-BCPP-BF showed an approximately 50% reduction by acetaminophen at the same dose of 300 mg/kg in rats, which are more resistant to acetaminophen than mice [31, 32].

Dysfunctions in mitochondrial oxidative processes after toxic doses of acetaminophen have been observed in rodents since the 1980s [33]. Early findings suggested that rats were more resistant to acetaminophen toxicity than mice, leading to the assumption that rats were not human-relevant species as an acetaminophen liver injury model [31, 32]. A recent study indicated that although overall acetaminophen metabolism was similar in both species, mitochondrial protein adducts were significantly lower in rats, which limits mitochondrial dysfunction and prevents oxidative stress and peroxynitrite formation in rats [33]. However, the present results demonstrated that MC-I activity in the rat liver measured using ^18^F-BCPP-BF was impaired by acetaminophen, even at a low dose of 100 mg/kg, a dose at which no apparent effects on hepatic function have been indicated in previous studies [24, 31, 32]. Dose-dependent reductions in ^18^F-BCPP-BF were observed at early as 2 h after acetaminophen dosing, at which the levels of AST, ALT, and bilirubin in plasma and histological liver damage remained unchanged. These results suggest that MC-I activity measured using ^18^F-BCPP-BF is more sensitive than conventional biochemical and histological parameters.

An overdose of acetaminophen induces acute hepatotoxic and nephrotoxic effects with massive hepatic centrilobular necrosis and acute renal failure, respectively, while there is evidence to show that the molecular basis of nephrotoxicity may differ from that of hepatotoxicity because NAC protects against hepatotoxicity, but not nephrotoxicity [3]. The present study also revealed dose range differences in acetaminophen-induced reductions in the uptake of ^18^F-BCPP-BF between the liver and kidney. Thus, a decrease in the uptake of ^8^F-BCPP-BF by the liver was observed, even with the low dose of 100 mg/kg, whereas a reduction in its uptake by the kidney required the administration of acetaminophen at higher doses of 300 mg/kg and more.

The increased generation of ROS within the mitochondrial electron transport chain is considered to be involved in pathogenic and drug-induced hepatitis and nephritis. In early stage of diabetes, mitochondrial fatty acid oxidation was found to be the source of increased net ROS production in the kidney tubule of the rat [34]. Although cisplatin is a widely used chemotherapeutic agent for cancers, its clinical use is limited due to its nephrotoxicity including acute kidney injury with elevated serum creatinine and BUN levels [35]. The mechanisms underlying cisplatin-induced nephrotoxicity remain unclear; it has been shown to induce impairments in energy metabolism and apoptosis in the mitochondria of the rat kidney [35]. Collectively, these findings and the present results indicate the potential of PET imaging of MC-I to become a useful diagnostic technique for drug-induced hepatotoxicity and nephrotoxicity.

One limitation of the present study was that ^18^F-BCPP-BF resulted in the slightly greater accumulation of radioactivity in bone with time, suggesting its instability with defluorination [19]. A common mechanism underlying ^18^F-fluoride formation is P-450-mediated hydroxylation occurred at ^18^F-fluorine-substituted carbon followed by spontaneous elimination [36]. However, significant bone uptake occurred slowly at the later phase of 60 min after the ^18^F-BCPP-BF injection [19], and the present study confirmed that imaging of kidney and liver with ^18^F-BCPP-BF required only 30 min after the injection. In addition, clinical assessment in human [37] as well as our previous animal study on mice [38] demonstrated that the injection of ^18^F-fluoride for bone scans did not accumulate in the kidney or liver, but strongly accumulated in the bladder. These results indicate that ^18^F-BCPP-BF is applicable to the quantitative imaging of MC-I activity in the renal cortex and liver.

## Conclusions

In conclusion, the present study demonstrated that PET imaging of MC-I has potential as a useful index for the non-invasive detection of hepatic and renal dysfunctions induced by an overdose of acetaminophen in the living body. These results suggested that, besides pathogenic hepatitis and nephritis, PET imaging of MC-I using ^18^F-BCPP-BF will become a useful diagnostic technique for hepatotoxicity and nephrotoxicity induced by other kinds of drugs such as cisplatin discussed above. In addition to diagnoses, PET imaging of MC-I may also be useful for monitoring the therapeutic effects of candidate agents for hepatic and nephritic diseases in vivo.
